# Long-Term Survival in Patients With Relapsed/Refractory Advanced Renal Cell Carcinoma Treated With Tivozanib: Analysis of the Phase III TIVO-3 Trial

**DOI:** 10.1093/oncolo/oyad348

**Published:** 2024-01-23

**Authors:** Kathryn E Beckermann, Aviva G Asnis-Alibozek, Michael B Atkins, Bernard Escudier, Thomas E Hutson, Vijay Kasturi, David F McDermott, Sumanta K Pal, Camillo Porta, Brian I Rini, Elena Verzoni

**Affiliations:** Division of Hematology Oncology, Vanderbilt-Ingram Cancer Center, Nashville, TN, USA; Clinical Development and Medical Affairs, AVEO Oncology, Boston, MA, USA; Department of Medical Oncology, Georgetown Lombardi Comprehensive Cancer Center, Washington, DC, USA; Department of Medical Oncology, Gustave Roussy, Villejuif, France; Texas A&M College of Medicine, Bryan, TX, USA; Clinical Development and Medical Affairs, AVEO Oncology, Boston, MA, USA; Department of Medicine, Beth Israel Deaconess Medical Center, Dana-Farber Cancer Institute/Harvard Cancer Center, Boston, MA, USA; Department of Medical Oncology and Therapeutics, City of Hope Comprehensive Cancer Center, Duarte, CA, USA; Interdisciplinary Department of Medicine, University of Bari Aldo Moro and Policlinico Consorziale di Bari, Bari, Italy; Division of Hematology Oncology, Vanderbilt-Ingram Cancer Center, Nashville, TN, USA; Department of Medical Oncology, Fondazione IRCCS Istituto Nazionale Tumori, Milan, Italy

**Keywords:** renal cell, carcinoma, long-term progression free survival, relapsed kidney cancer, tivozanib, vascular endothelial growth factor

## Abstract

**Background:**

Tivozanib is an oral vascular endothelial growth factor receptor (VEGFR) tyrosine kinase inhibitor (TKI) with efficacy in advanced renal cell carcinoma (RCC). Long-term exploratory analyses from the TIVO-3 trial in relapsed/refractory (R/R) RCC including patients (26%) with prior immuno-oncology (IO) therapy are reported.

**Methods:**

Patients with R/R advanced RCC that progressed with 2 or 3 prior systemic therapies (≥1 VEGFR TKI) were randomized to tivozanib 1.5 mg QD or sorafenib 400 mg BID, stratified by IMDC risk and previous therapy. Safety, investigator-assessed long-term progression-free survival (LT-PFS), and serial overall survival (OS) were assessed.

**Results:**

Mean time on treatment was 11.0 months with tivozanib (*n* = 175) and 6.3 months with sorafenib (*n* = 175). Fewer grade ≥3 treatment-related adverse events occurred with tivozanib (46%) than sorafenib (55%). Dose modification rates were lower with tivozanib than sorafenib across age/prior IO subgroups; prior IO therapy did not impact dose reductions or discontinuations in either arm. Landmark LT-PFS rates were higher with tivozanib (3 years: 12.3% vs 2.4%; 4 years: 7.6% vs 0%). After 22.8 months mean follow-up, the OS HR was 0.89 (95% CI, 0.70-1.14); when conditioned on 12-month landmark PFS, tivozanib showed significant OS improvement over sorafenib (HR, 0.45; 95% CI, 0.22-0.91; 2-sided *P* = .0221).

**Conclusions:**

Tivozanib demonstrated a consistent safety profile and long-term survival benefit in patients with R/R advanced RCC who were alive and progression free at 12 months. These post hoc exploratory analyses of LT-PFS and conditional OS support a clinically meaningful improvement with tivozanib versus sorafenib in this advanced RCC population.

## Teaser

Tivozanib is an oral vascular endothelial growth factor receptor tyrosine kinase inhibitor with efficacy in advanced renal cell carcinoma (RCC). Long-term exploratory analyses from the TIVO-3 trial in relapsed/refractory RCC, including patients with prior immuno-oncology therapy, are reported.

**Implications for Practice**

Patients with relapsed/refractory (R/R) advanced renal cell carcinoma (RCC) need tolerable treatments that provide clinical benefit. In this post hoc analysis of the TIVO-3 trial, we evaluated long-term safety and survival with tivozanib versus sorafenib in R/R advanced RCC that progressed on 2-3 prior systemic therapies including a VEGFR TKI, with a notable proportion (26%) having received prior immuno-oncology therapy and a VEGFR TKI sequentially or in combination. Tivozanib demonstrated durable clinical benefit and safety across age groups and regardless of prior treatment. This exploratory analysis continues to support the use of tivozanib in the heavily pretreated advanced RCC population.

## Introduction

In the US in 2022, an estimated 79 000 new cases were diagnosed and 14 000 people died from kidney cancer.^1^ Renal cell carcinoma (RCC) accounts for 80%-90% of kidney malignancies.^2,3^ Most patients with RCC in the US are >65 years of age; the largest increase in incidence is among those aged ≥75 years.^4^ One in 5 patients with RCC have metastases at initial presentation,^5,6^ and one-third of patients with localized disease experience recurrence.^7,8^

RCC treatment has rapidly evolved with the development of targeted therapies and immuno-oncology (IO) therapies.^9^ IO therapies have demonstrated efficacy in RCC as monotherapies and in combination with other IO therapies or vascular endothelial growth factor receptor (VEGFR) tyrosine kinase inhibitors (TKIs).^10-12^ IO combination therapy has emerged as the frontline standard of care for patients with advanced RCC who require systemic therapy.^9^ The majority of patients ultimately experience disease progression and require subsequent treatments.^13^ These limitations highlight the need for safe and effective therapies in the refractory setting.

In the phase III METEOR trial, cabozantinib improved overall survival (OS) and progression-free survival (PFS) versus everolimus in patients with metastatic RCC (mRCC) who had disease progression with ≥1 VEGFR TKI, with 5% having also received prior IO therapy.^14^ In a phase II trial, lenvatinib plus everolimus showed clinical benefit in patients with mRCC whose disease progressed with 1 prior VEGF-targeted therapy.^15^ Recently, a single-arm, phase II trial and a retrospective study demonstrated that VEGFR TKIs (axitinib, pazopanib, sunitinib, and cabozantinib) have therapeutic activity in patients whose disease progressed with IO therapy and VEGF-targeted therapy.^16,17^

Tivozanib is a potent and selective inhibitor of VEGFR 1, 2, and 3, with a long half-life (4-5 days) that was designed to optimize VEGF blockade while minimizing off-target side effects, resulting in improved efficacy and less need for dose modifications than with other VEGFR inhibitors (eg, sorafenib).^18-20^ TIVO-3, which compared tivozanib with sorafenib, is the first phase III trial that prospectively evaluated long-term efficacy data in patients with advanced RCC whose disease was refractory to prior therapies, including a predefined subgroup of patients previously treated with IO therapy and VEGFR TKIs (26%).^13,20^ Treatment-related adverse events (TRAEs) were seen in 146 patients (84%) receiving tivozanib and 160 patients (94%) receiving sorafenib.^20^ Primary results demonstrated a median PFS of 5.6 months (95% CI, 5.29-7.33 months) with tivozanib versus 3.9 months (95% CI, 3.71-5.55 months) with sorafenib (hazard ratio [HR], 0.73; 95% CI, 0.56-0.94; 2-sided *P* = .016).^20^ In 2021, the US Food and Drug Administration approved tivozanib for adult patients with relapsed or refractory (R/R) advanced RCC following ≥2 prior systemic therapies based on results from TIVO-3.^21,22^

To better understand the population of patients likely to derive long-term benefit from tivozanib in a refractory setting, we evaluated long-term PFS (LT-PFS), serial OS, and OS conditioned on a clinically meaningful 12-month time point in patients from the TIVO-3 trial. Here, we report long-term safety and survival results from planned and exploratory post hoc analyses from TIVO-3.

## Patients and Methods

### Study Design and Treatment Regimen

TIVO-3 (NCT02627963) was a phase III, global, open-label, parallel-arm study comparing the safety and efficacy of tivozanib versus sorafenib in patients with R/R advanced RCC whose disease progressed with 2 or 3 prior VEGFR TKI systemic regimens, including a predefined subgroup who received prior IO therapy. Patients were randomized 1:1 to receive 1.5 mg of tivozanib hydrochloride (equivalent to 1.34 mg of tivozanib free base) once daily or 400 mg of sorafenib twice daily and stratified by International Metastatic RCC Database Consortium risk and prior therapy. For more details, see the Supplementary Methods.

### Eligibility Criteria

Eligibility criteria were previously reported.^20^ Briefly, patients were ≥18 years of age, had confirmed mRCC with a clear-cell component, and experienced disease progression with 2 or 3 prior systemic regimens. Key exclusion criteria included substantial cardiovascular disease or active brain metastases.

### Endpoints and Assessment

Endpoints were previously described.^20^ Briefly, the primary endpoint was PFS; secondary endpoints included OS and safety. Exploratory post hoc analyses described here included LT-PFS, serial OS, and a 12-month PFS-conditioned OS assessed by the investigator (INV) at the final data cutoff (May 24, 2021). PFS was defined as the time from randomization to first documented tumor progression according to Response Evaluation Criteria in Solid Tumors version 1.1 or death. OS was defined as the time from randomization to death. PFS-conditioned OS was defined as the OS time for the subpopulation alive and progression free at 12 months. Safety was assessed in patients stratified by age groups and prior IO therapy based on the May 2020 data cutoff. For more details, see the Supplementary Methods.

### Statistical Analysis

Results included findings from the intention-to-treat (ITT) population. Tivozanib and sorafenib safety profiles were compared in patients stratified by age group (<65, 65-74, and ≥75 years) and exposure to prior IO therapy. Safety assessments and baseline characteristics were reported using descriptive statistics.

Survival analyses included censoring for missing assessments and treatment discontinuations in the absence of progressive disease. In these exploratory post hoc analyses, which were based on fixed time points, the mean provides a better estimate of changes over time, allowing determination of the potential long-term survival benefit.^23^ LT-PFS landmark values at 6, 12, 18, 24, 30, 36, 42, and 48 months were assessed at the final data cutoff (May 24, 2021), 5 years after the first patient was enrolled. Cox proportional hazards models and log-rank statistics were used to estimate the HR (95% CI) for PFS using prespecified and exploratory analyses; odds ratios were reported for landmark time points of LT-PFS ≤36 months.

To evaluate the effects of OS maturation, ITT analyses of Cox proportional hazards models and log-rank statistics were used to estimate HRs and 95% CIs for OS at prespecified (2 years after last patient in 251 events) and exploratory extended follow-up (270 events) time points. At the final data cutoff, conditional analyses of Cox proportional hazards models and stratified log-rank statistics from patients achieving 12-month PFS in either group were used to estimate the HR and 95% CI for OS. Conditional OS was assessed until death or censoring at the date of last follow-up. Data from patients who died or whose follow-up interval ended before the landmark time were excluded.

## Results

### Patients and Baseline Characteristics

As of May 24, 2021, 350 patients with advanced RCC were randomized to receive tivozanib or sorafenib. The ITT population included 175 patients in each arm, and the safety population included 173 patients in the tivozanib arm and 170 in the sorafenib arm (Supplementary Fig. S1). Baseline demographic and disease characteristics were balanced between groups and consistent with an advanced RCC population (Supplementary Table S1). All patients previously received a VEGFR TKI, with one-quarter having received a prior IO therapy and a VEGFR TKI sequentially or in combination. In both arms, the median time from initial diagnosis to randomization was 50 months.^20^

### Safety

**Figure 1.**
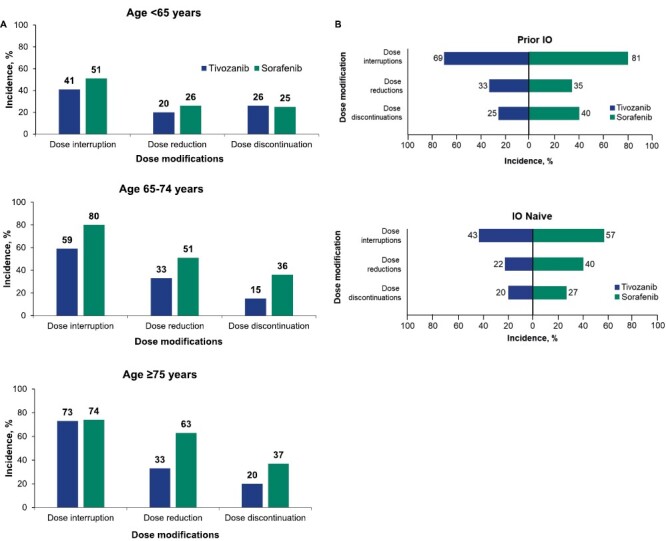
Dose modifications by age and prior IO therapy status. Dose interruption, dose reduction, and dose discontinuation for patients treated with tivozanib or sorafenib by age (<65, 65-74, and ≥75 years; **A**) and IO status (**B**). Data cutoff: May 2020. Abbreviation: IO, immuno-oncology.

The safety of tivozanib compared with that of sorafenib was assessed in subgroups stratified by age (<65 [55%], 65-74 [35%], and ≥75 [10%] years) and prior IO therapy (27% vs 73% without). Patients treated with tivozanib remained on treatment longer (mean, 11.0 months; range, 0.1-36.9 months) than patients treated with sorafenib (mean, 6.3 months; range, 0.2-36.1 months). Treatment duration did not depend on age or prior IO therapy subgroup (Supplementary Table S2). The dose modification rate was lower with tivozanib than with sorafenib across subgroups (Fig. 1). Among patients ≥75 years of age, the rates of dose reductions and treatment discontinuations were lower with tivozanib (33% and 20%, respectively) than with sorafenib (63% and 37%, respectively) (Fig. 1A). There were no clinically significant differences in dose reductions or discontinuations based on prior IO therapy in either arm (Fig. 1B); however, dose holds were >20% higher in patients with versus without prior IO therapy in both treatment arms. With longer follow-up, no treatment-related deaths occurred with tivozanib or sorafenib.

Grade ≥3 TRAEs attributed to VEGFR TKI class effects were reported in 46% and 55% of patients treated with tivozanib and sorafenib, respectively, with the most common (>5% incidence in either arm) being hypertension, diarrhea, fatigue, asthenia, rash, and palmar-plantar erythrodysesthesia (Supplementary Table S3). Hypertension was reported in 20% of patients treated with tivozanib and in 14% treated with sorafenib and rates of asthenia were similar at 5% and 4%, respectively. All other common class-effect grade ≥3 TRAEs were observed more frequently with sorafenib.

**Figure 2.**
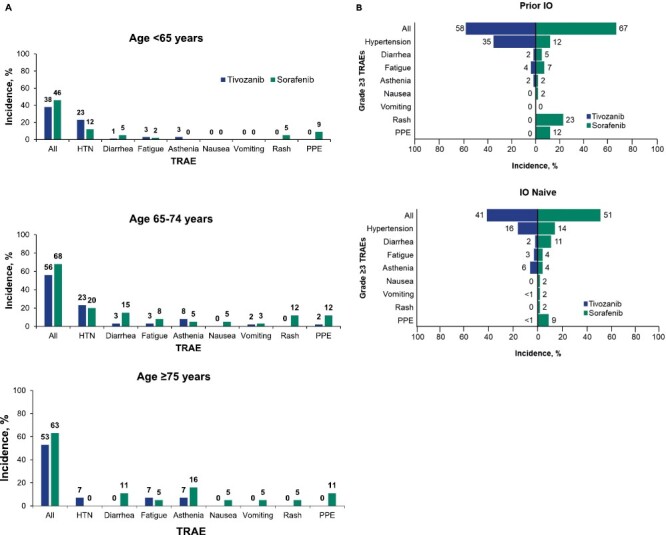
Grade ≥3 TRAEs attributed to VEGFR TKI class effects by age and prior IO therapy status. Grade ≥3 TRAEs for patients treated with tivozanib or sorafenib by age (<65, 65-74, and ≥75 years; **A**) and IO therapy status (**B**). Data cutoff: May 2020. TRAE grading was performed using the criteria from the National Cancer Institute Common Terminology Criteria for Adverse Events (version 4.03). Overall grade ≥3 TRAEs and grade ≥3 TRAEs attributed to VEGFR TKI class effects were reported. Abbreviations: IO, immuno-oncology; PPE, palmar-plantar erythrodysesthesia; TKI, tyrosine kinase inhibitor; TRAE, treatment-related adverse event; VEGFR, vascular endothelial growth factor receptor.

When grade ≥3 TRAEs attributed to VEGFR TKI class effects were analyzed in patients stratified by age, the safety profile was generally retained across age subgroups, with a higher overall incidence of TRAEs in patients aged 65-74 years (56% and 68%) and ≥75 years (53% and 63%) than in those aged <65 years (38% and 46%) in the tivozanib and sorafenib arms, respectively (Fig. 2A). In patients aged ≥75 years, hypertension was uncommon (tivozanib [7%] and sorafenib [0%]) compared with patients aged <65 years (tivozanib [23%] and sorafenib [12%]). Respective rates of asthenia (7% and 16%) and fatigue (7% and 5%) in patients aged ≥75 years in the tivozanib and sorafenib arms were generally higher than in younger patients (<65 years: asthenia [3% and 0%] and fatigue [3% and 2%]).

When the incidence of grade ≥3 TRAEs attributed to VEGFR TKI class effects was assessed for patients stratified by prior IO therapy, overall rates of grade ≥3 TRAEs were higher in both arms treated with prior IO therapy (tivozanib [58%] and sorafenib [67%]) compared with those without prior IO therapy (tivozanib [41%] and sorafenib [51%]; Fig. 2B). Hypertension occurred more frequently with tivozanib and prior IO therapy (35% vs 16% in IO-naive patients), whereas rash occurred more frequently with sorafenib and prior IO exposure (23% vs 2% in IO-naive patients).

### Efficacy

#### Investigator-Assessed LT-PFS

**Figure 3.**
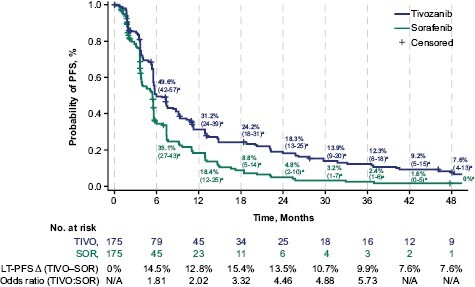
Landmark rates (95% CI) of LT-PFS in TIVO-3: tivozanib versus sorafenib. The INV-assessed PFS HR with extended follow-up was 0.624 (95% CI, 0.49-0.79) and favored tivozanib over sorafenib (2-sided, log-rank *P* < .0001). HR was obtained from a Cox hazards regression model, and *P* values were 2-sided log-rank tests. ^a^Percentage (95% CI); ^b^odds ratio not calculated at months 42 and 48 due to insufficient number at risk. Abbreviations: ∆, absolute difference; HR, hazard ratio; INV, investigator; LT-PFS, long-term progression-free survival; SOR, sorafenib; TIVO, tivozanib.

The INV-assessed PFS HR analyzed with extended follow-up (data cutoff: May 24, 2021) favored tivozanib over sorafenib (HR, 0.624; 95% CI, 0.49-0.79; Fig. 3) and was comparable to the independent review committee–assessed PFS HR reported at the earlier October 2018 data cutoff (HR, 0.672; 95% CI, 0.52-0.87).^24^ In this post hoc exploratory analysis, we assessd the proportion of patients who achieved LT-PFS at regular intervals up to 48 months after treatment initiation. Landmark INV-assessed LT-PFS rates up to 48 months are shown in Fig. 3; rates were consistently higher with tivozanib versus sorafenib, with differences between arms of 12.8% (odds ratio [OR], 2.02), 9.9% (OR, 5.73), and 7.6% (OR, not available) at 12, 36, and 48 months, respectively (Fig. 3). With tivozanib, point estimates for the probability of PFS were higher than those with sorafenib at each subsequent year. After 36 months, patients treated with tivozanib were >5 times more likely to experience LT-PFS than patients receiving sorafenib. At 48 months, the number of patients with PFS data available in both arms was too low to support a meaningful analysis. In patients stratified by subgroups, INV-assessed LT-PFS rates were higher in the tivozanib arm than in the sorafenib arm (Table 1).

**Table 1.** Landmark long-term PFS by subgroup per investigator.

Subgroup	TIVO, n	SOR, n	12-month PFS, %		24-month PFS, %		36-month PFS, %		48-month PFS, %	
			TIVO	SOR	TIVO	SOR	TIVO	SOR	TIVO	SOR
No. at risk	175	175	45	23	25	6	16	3	9	1
IMDC										
Favorable	34	36	44.0	33.6	24.0	8.4	**17.1**	0	NE	NE
Intermediate	109	105	30.9	18.7	19.9	5.3	14.8^a^	4.0	11.9	0
Poor	32	34	18.3	NE	7.3	NE	NE	NE	NE	NE
Prior treatment										
Any immunotherapy	47	44	27.0	18.6	19.1	3.7	9.8^a^	NE	6.5	NE
TKI-TKI only	79	80	31.6	9.8	18.6	2.0	13.5	NE	NE	NE
No immunotherapy	128	131	32.7	18.3	18.1	5.1	13.0	2.0	7.9	NE
Sex										
Female	49	47	32.5	12.3	25.0	0	**17.5** ^a^	NE	12.5	NE
Male	126	128	30.7	20.3	15.6	6.4	10.1	3.2	5.4	NE
Age										
<65 y	98	95	26.4	13.8	12.9	4.2	10.0	2.8	5.7	NE
≥65 y	77	80	37.3	24.2	24.9	5.6	**15.3**	NE	9.9	NE
ECOG PS^b^										
0	85	83	39.0	17.4	20.5	6.3	**16.0**	3.2	10.9	NE
≥1	88	86	23.3	19.7	NE	3.3	8.7	NE	4.4	NE
Geographic region										
Europe	144	148	31.0	18.6	15.7	4.2	10.5	1.7	6.9	NE
North America	31	27	32.3	14.3	NE	NE	**16.5** ^**c**^	NE	11.0	NE
Prior lines of treatment										
2	108	104	32.4	21.0	17.2	5.6	12.3	1.4	6.1	NE
≥3	67	71	29.0	14.8	19.9	3.7	12.2^a^	NE	9.8	NE

Data cutoff: May 24, 2021. Bold-faced numbers indicate values ≥15% INV-assessed LT-PFS at 36 months.

^a^30-month value.

^b^Excludes 8 patients with unknown performance status.

^c^42-month value.

Abbreviations: ECOG PS, Eastern Cooperative Oncology Group performance status; IMDC, International mRCC Database Consortium; INV, investigator; LT-PFS, long-term progression-free survival; NE, not estimable; SOR, sorafenib; TIVO, tivozanib; TKI, tyrosine kinase inhibitor.

#### Serial OS With Extended Data (ITT Population)

**Figure 4.**
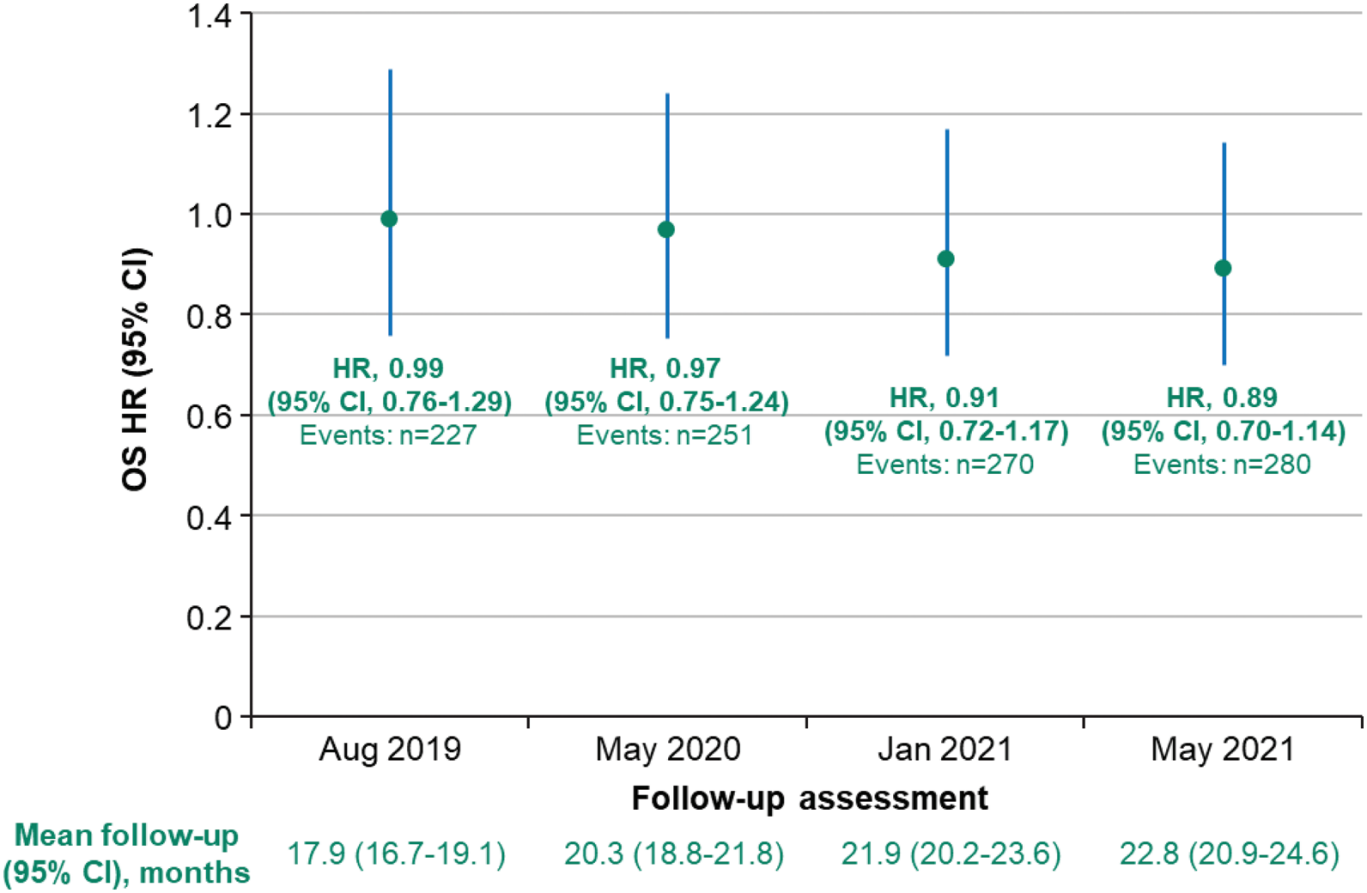
Serial OS with extended follow-up. To evaluate for effects of maturation of OS, Cox proportional hazards and log-rank statistics were used to estimate HR (95% CI) for OS using prespecified (2 years after last patient in, August 2019; 251 events, May 2020) and exploratory (extended follow-up: 270 events, January 2021; database closure, May 24, 2021) ITT analyses. HR, hazard ratio; ITT, intention to treat; OS, overall survival.

At 2 years after the last patient in (mean follow-up: 17.9 months; data cutoff: August 2019), 65% of patients had experienced an event, and the OS HR was 0.99 (95% CI, 0.76-1.29; Fig. 4). At final database closure (mean follow-up: 22.8 months; data cutoff: May 24, 2021) and after the occurrence of 80% of planned events, the OS HR was 0.89 (95% CI, 0.70-1.14; 2-sided *P* = .3533) with a median OS that was lower with tivozanib (16.4 months; 95% CI, 13.4-21.9 months) than with sorafenib (19.1 months; 95% CI, 14.9-24.2 months; Table 2).

**Table 2.** Unconditioned (ITT population) and landmark PFS-conditioned overall survival in TIVO-3.

Population	Group	At risk, *n*	Events, *n*	Median OS (95% CI), mo	HR (95% CI)	Stratified log-rank *P*-value
Unconditioned (ITT population)	Tivozanib	175	138	16.4 (13.4-21.9)	0.89 (0.70-1.14)	.3533
	Sorafenib	175	142	19.1 (14.9-24.2)		
Conditioned on PFS ≥ 12 months	Tivozanib	45	25	48.3 (32.8-NR)	0.45 (0.22-0.91)	.0221
	Sorafenib	23	17	32.8 (27.6-50.0)		
Conditioned on PFS ≥ 18 months	Tivozanib	34	8	54.3(44.9-NR)	0.46 (0.15-1.39)	.1617
	Sorafenib	11	5	50.0(32.4-NR)		

Abbreviations: HR, hazard ratio; ITT, intention to treat; NR, not reached; OS, overall survival; PFS, progression-free survival.

#### Landmark PFS-Conditioned OS

**Figure 5.**
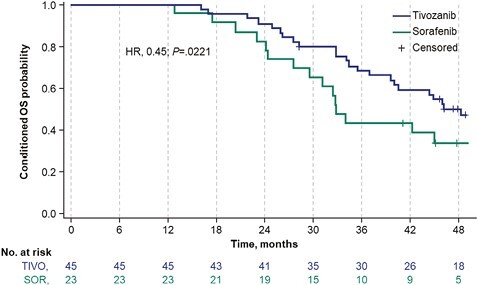
Kaplan-Meier survival curve of conditional OS in patients with 12-month PFS. The Kaplan-Meier survival curves for tivozanib and sorafenib groups conditioned on 12-month PFS. Conditional analyses of Cox proportional hazards models and stratified log-rank statistics, using data from patients achieving 12-month PFS in either group, were used to estimate the hazard ratio. Data cutoff: May 24, 2021. Abbreviations: HR, hazard ratio; OS, overall survival; PFS, progression-free survival; SOR, sorafenib; TIVO, tivozanib.

In a post hoc analysis, when OS was conditioned on a clinically relevant 12-month landmark PFS time point, a statistically significant improvement in OS was observed with tivozanib compared with sorafenib (HR, 0.45; 95% CI, 0.22-0.91; 2-sided *P* = .0221). Median OS was 48.3 months (95% CI, 32.8 months to not reached) with tivozanib versus 32.8 months (95% CI, 27.6-50.0 months) with sorafenib (Table 2). The Kaplan-Meier survival curves demonstrated a rapid separation shortly after the 1-year time point that remained consistent over time (Fig. 5).

## Discussion

With 4 years of follow-up in TIVO-3, tivozanib demonstrated a long-term safety and LT-PFS benefit versus sorafenib in patients with R/R advanced RCC, establishing consistent durability of response with tivozanib. The INV-assessed PFS rate analyzed with extended follow-up was consistent with the independent review committee–assessed PFS rate in the primary analysis,^20^ providing further evidence of the clinical benefit of tivozanib compared with sorafenib. Additional exploratory post hoc analysis, including serial OS with extended follow-up, suggests improvement of survival over time though changes in the HR were not significant. These data, together with the results from the 12-month conditional OS analysis, suggest that there is a clinically meaningful population of patients who experienced long-term benefit with tivozanib treatment.

This post hoc extended follow-up analysis adds more evidence to support the clinical value of tivozanib in the third- or fourth-line refractory setting. TKIs such as tivozanib and sorafenib are noncurative, underscoring the importance of measuring LT-PFS. If cure is not achievable, long-term clinical benefit, as assessed by LT-PFS, therapeutic index, and quality of life are ideal measures of the value of anticancer agents.^9^ Additionally, these exploratory analyses aim to further our understanding of the durability of tivozanib’s clinical benefit, recognizing that OS analysis can vary by region based on the availability of subsequent treatment options.^25^

To our knowledge, this study represents the first use of conditional OS to demonstrate a comparative clinical advantage of a VEGFR TKI. When conditioned based on 12-month landmark PFS, tivozanib showed a significant improvement in OS over sorafenib (HR, 0.45; 95% CI, 0.22-0.91; 2-sided *P* = .0221). In addition, a clinically relevant proportion of patients, 9.9% and 7.6%, were alive and progression free at 3 and 4 years, respectively, after initiating tivozanib, further supporting the clinical benefit of tivozanib because many patients with R/R mRCC do not survive to these time points. Moreover, the higher probability of PFS observed with tivozanib versus sorafenib at 3 and 4 years was seen across subgroups stratified by age or prior IO therapy.

Tivozanib demonstrated a favorable safety profile, as indicated by the fewer dose adjustments necessary with tivozanib compared with sorafenib in patients with R/R advanced RCC. Patients in the tivozanib arm remained on treatment longer than patients treated with sorafenib. Additionally, other than hypertension and asthenia, common class-effect grade ≥3 TRAEs were observed more frequently with sorafenib. Interestingly, patients with prior IO therapy experienced a higher percentage of hypertension with tivozanib and rash with sorafenib. Additionally, the frequency of dose holds was >20% higher in patients with prior IO therapy compared with patients without prior IO therapy. To our knowledge, this is the first report of increased AE incidence based on prior IO treatment in RCC. In a retrospective study of patients with melanoma who received programmed cell death 1 (PD-1) inhibitor and cytotoxic T-lymphocyte-associated protein 4 (CTLA-4) inhibitor therapy, there was an increase in systolic blood pressure when the use of anti-hypertensive agents and increased pain were controlled.^26^ Further studies will be needed to confirm this potential for an increased incidence of hypertension following treatment with IO therapy.

The therapeutic index of tivozanib and the long-term efficacy results observed in TIVO-3 and further explored by this post hoc exploratory analysis support its use as a subsequent therapy option. Tivozanib is being evaluated in combination with the PD-1 inhibitor nivolumab versus tivozanib monotherapy in patients with advanced RCC who received prior IO therapy in the phase III TiNivo-2 study (NCT04987203).^27^

These post hoc exploratory analyses of the TIVO-3 study have several limitations. The sample size and small numbers of patients in the sorafenib arm who were at risk for progression at 2 (*n* = 6) and 3 (*n* = 3) years and alive at 3 (*n* = 10) and 4 (*n* = 5) years limited long-term statistical comparisons. Moreover, we recognize that the field of kidney cancer therapy is moving rapidly and while these post hoc analyses were initiated to gather further information on patients treated with prior IO therapy, the timing of this study did not allow for inclusion of many patients treated with what is now standard anti–PD-1 backbone combination therapy with a VEGFR TKI or anti–CTLA-4, but instead also included patients who received a prior anti–PD-1 and a VEGFR TKI sequentially. However, these results from the TIVO-3 study demonstrate that patients with prior IO therapy exposure can benefit from tivozanib in the refractory setting. Furthermore, we recognize the limitation of the selected 12-month conditional OS analysis time point. Although this was based on a clinically meaningful time point in a refractory RCC setting, it required patients to be alive 12 months after treatment initiation, therefore limiting the number of patients available for analysis. Finally, since all the long-term analyses were exploratory, additional phase III, prospective studies are needed to confirm the treatment effects reported here.

## Conclusions

The current exploratory analysis of long-term survival in patients with R/R advanced RCC demonstrated continued clinical benefit with tivozanib versus sorafenib over a 4-year follow-up period in a clinically meaningful population of patients who were alive and progression free at 12 months. Long-term benefits were seen across all age groups and regardless of prior therapies. These findings support using tivozanib in the treatment paradigm for patients with R/R advanced RCC, regardless of age or prior immunotherapy exposure.

## Supplementary Material

oyad348_suppl_Supplementary_Methods

oyad348_suppl_Supplementary_Figures_S1

oyad348_suppl_Supplementary_Tables_S1-S3

## Data Availability

De-identified individual participant data will be available immediately after publication to applicants who provide a sound proposal to the AVEO Oncology Research Unit Data Access Committee.

## Clinical Trial Information

Clinical trial registration: NCT02627963.

## Prior Presentations

Presented at the 2022 ASCO Genitourinary Cancer Symposium; San Francisco, CA, February 17-19, 2022; the 2022 ASCO Annual Meeting, Chicago, IL, and Virtual, June 3-7, 2022; and the Society for Immunotherapy of Cancer (SITC) 2021, Washington, DC, and Virtual, November 10-14, 2021.
