# Supplementary material for: Long-Term Survival in Patients With Relapsed/Refractory Advanced Renal Cell Carcinoma Treated With Tivozanib: Analysis of the Phase III TIVO-3 Trial
Source: Oncologist. 2024 Jan 23;29(3):254–62. doi: 10.1093/oncolo/oyad348 (PMC10911910; doi:10.1093/oncolo/oyad348)
Supplement: oyad348_suppl_Supplementary_Methods [file oyad348_suppl_supplementary_methods.docx]

**Supplementary Methods**

***Study design and treatment regimen***

Patients were randomized 1:1 to receive 1.5 mg of tivozanib hydrochloride (equivalent to 1.34 mg of tivozanib free base) once daily in 4-week cycles comprising 21 days on treatment followed by 7 days off treatment or 400 mg of sorafenib twice daily continuously (with 1 cycle comprising 4 weeks of treatment). Patients were stratified by prior therapy (2 prior tyrosine kinase inhibitors [TKIs], a prior immuno-oncology [IO] therapy and TKI, or a prior TKI and any other systemic agent) and by International Metastatic Renal Cell Carcinoma Database Consortium prognostic score. Dose reductions (to 1.0 mg per day for tivozanib or 400 mg per day for sorafenib) were allowed for patients with treatment-related adverse events of grade 3 or higher, and dose interruptions were allowed for the management of persistent adverse events. Patients received study treatment until disease progression was confirmed by the independent radiology review committee according to RECIST v1.1) or unacceptable toxicity.

This trial was approved by the institutional review board or ethics committee at each center and was conducted in accordance with Good Clinical Practice guidelines and the Declaration of Helsinki. The patients discussed in this manuscript have given written informed consent to publication of their case details.

***Endpoints and assessments***

Adverse events were coded using the Medical Dictionary for Regulatory Activities (MedDRA) version 19.1 or later. TRAEs were graded according to National Cancer Institute Common Terminology Criteria for Adverse Events version 4.03. A data monitoring committee reviewed the safety data of the study.
