# Supplementary figures and images for: Long-Term Survival in Patients With Relapsed/Refractory Advanced Renal Cell Carcinoma Treated With Tivozanib: Analysis of the Phase III TIVO-3 Trial
Source: Oncologist. 2024 Jan 23;29(3):254–62. doi: 10.1093/oncolo/oyad348 (PMC10911910; doi:10.1093/oncolo/oyad348)

**Supplementary Figure 1. CONSORT Diagram for the phase III TIVO-3 trial**

| **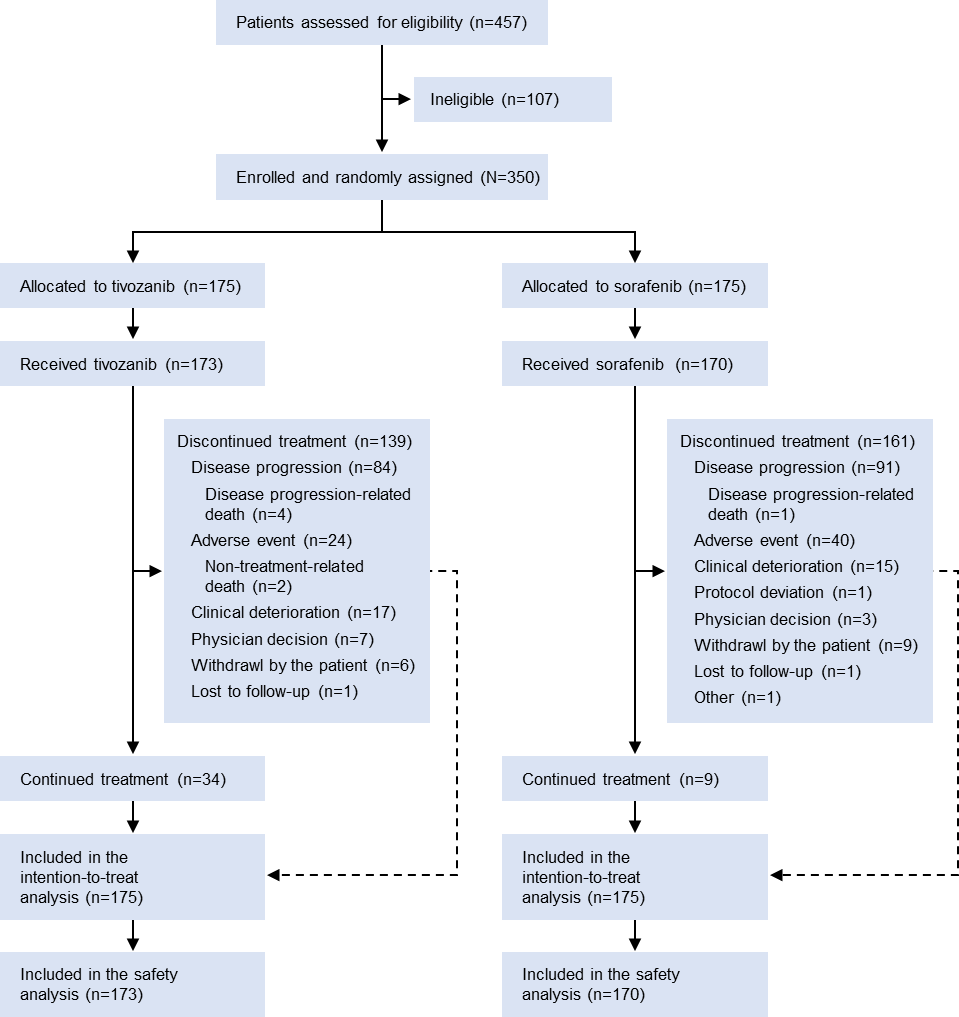** |
| --- |

Supplement: oyad348_suppl_Supplementary_Figures_S1 [file oyad348_suppl_supplementary_figures_s1.docx]
