# Supplementary material for: Long-Term Survival in Patients With Relapsed/Refractory Advanced Renal Cell Carcinoma Treated With Tivozanib: Analysis of the Phase III TIVO-3 Trial
Source: Oncologist. 2024 Jan 23;29(3):254–62. doi: 10.1093/oncolo/oyad348 (PMC10911910; doi:10.1093/oncolo/oyad348)
Supplement: oyad348_suppl_Supplementary_Tables_S1-S3 [file oyad348_suppl_supplementary_tables_s1-s3.docx]

**Supplementary Table 1. Demographic and baseline characteristics in the ITT population**

| **Characteristics** | **Tivozanib (n=175)** | **Sorafenib (n=175)** |
| --- | --- | --- |
| **Age, mean (range), y** | 62 (34-88) | 63 (30-90) |
| **Sex, n (%)** |  |  |
| **Male** | 126 (72) | 128 (73) |
| **Female** | 49 (28) | 47 (27) |
| **Race, n (%)** |  |  |
| **White** | 165 (94) | 167 (95) |
| **Asian** | 2 (1) | 2 (1) |
| **Black or African American** | 0 | 2 (1) |
| **Other/unknown** | 8 (5) | 4 (2) |
| **Pathological diagnosis, n (%)** |  |  |
| **Clear cell** | 165 (94) | 160 (91) |
| **Clear-cell component** | 9 (5) | 9 (5) |
| **Other^a^** | 1 (1) | 5 (3) |
| **IMDC risk category, n (%)** |  |  |
| **Favorable** | 34 (19) | 36 (21) |
| **Intermediate** | 109 (62) | 105 (60) |
| **Poor** | 32 (18) | 34 (19) |
| **No. of previous systemic therapies, n (%)** |  |  |
| **2** | 108 (62) | 104 (59) |
| **3** | 67 (38) | 71 (41) |
| **Previous therapies, n (%)** |  |  |
| **2 VEGFR TKIs** | 79 (45) | 80 (46) |
| **Checkpoint inhibitor and  VEGFR TKI^b^** | 47 (27) | 44 (25) |
| **VEGFR TKI and other systemic agent^c^** | 49 (28) | 51 (29) |
| **Time from initial diagnosis, median (range), mo^d^** | 50 (10-347) | 50 (9-224) |
| **Time from most recent relapse, median (range), mo^e^** | 1 (<1-121) | 1 (<1-87) |

IMDC, International Metastatic Renal Cell Carcinoma Database Consortium; VEGFR, vascular endothelial growth factor receptor; ITT, intention to treat; TKI, tyrosine kinase inhibitor.

^a^ One patient treated with sorafenib had missing pathological diagnosis information. ^b^ Therapies were sequential or in combination. ^c^ Of the 100 patients who received previous VEGFR TKI and another systemic agent, 43 received everolimus, 31 received interleukin-2, 19 received interferon, and 3 received temsirolimus. ^d^ Time from initial diagnosis (n=147 for tivozanib and n=144 for sorafenib) was calculated using randomization date as an anchor point. ^e^ Time from most recent relapse (n=164 for tivozanib and n=166 for sorafenib).

**Supplementary Table 2. Dose modifications by age and prior immuno-oncology therapy status**

|  | | **Drug exposure, mean cycles, n** | **Dose interruption, %** | **Dose reduction, %** | **Dose discontinuation, %** |
| --- | --- | --- | --- | --- | --- |
| **All** | Tivozanib (n=173) | 11.9 | 48 | 24 | 21 |
|  | Sorafenib (n=170) | 6.7 | 64 | 39 | 30 |
| **Age <65 y** | Tivozanib (n=97) | 10.1 | 41 | 20 | 26 |
|  | Sorafenib (n=92) | 6.7 | 51 | 26 | 25 |
| **Age 65-74 y** | Tivozanib (n=61) | 15.0 | 59 | 33 | 15 |
|  | Sorafenib (n=59) | 6.6 | 80 | 51 | 36 |
| **Age ≥75 y** | Tivozanib (n=15) | 11.2 | 73 | 33 | 20 |
|  | Sorafenib (n=19) | 7.3 | 74 | 63 | 37 |
| **Prior IO** | Tivozanib (n=48) | 12.3 | 69 | 33 | 25 |
|  | Sorafenib (n=43) | 5.5 | 81 | 35 | 40 |
| **No. of prior IO** | Tivozanib (n=125) | 11.7 | 43 | 22 | 20 |
|  | Sorafenib (n=127) | 7.2 | 57 | 40 | 27 |

IO, immuno-oncology.

**Supplementary Table 3. Grade ≥3 TRAEs attributed to VEGFR TKI class effects in all patients**

|  | **Grade ≥3 TRAEs, incidence n (%)** | |
| --- | --- | --- |
| **Preferred term** | **Tivozanib (n=173)** | **Sorafenib (n=170)** |
| All TRAEs | 80 (46) | 94 (55) |
| Hypertension | 35 (20) | 23 (14) |
| Diarrhea | 3 (2) | 16 (9) |
| Fatigue | 6 (4) | 8 (5) |
| Asthenia | 8 (5) | 6 (4) |
| Nausea | 0 | 4 (2) |
| Vomiting | 1 (1) | 3 (2) |
| Rash | 0 | 13 (8) |
| PPE | 1 (1) | 17 (10) |

PPE, palmar-plantar erythrodysesthesia; VEGFR, vascular endothelial growth factor receptor; TKI, tyrosine kinase inhibitor; TRAE, treatment-related adverse event.
